# Putative Role of Vitamin D for COVID-19 Vaccination

**DOI:** 10.3390/ijms22168988

**Published:** 2021-08-20

**Authors:** Sheng-Kang Chiu, Kuo-Wang Tsai, Chia-Chao Wu, Cai-Mei Zheng, Chung-Hsiang Yang, Wan-Chung Hu, Yi-Chou Hou, Kuo-Cheng Lu, You-Chen Chao

**Affiliations:** 1 Division of Infection, Department of Medicine, Taipei Tzu Chi Hospital, Buddhist Tzu Chi Medical Foundation, New Taipei City 231, Taiwan; csk33kimo@hotmail.com; 2 School of Medicine, Tzu Chi University, Hualien 970, Taiwan; 3 Division of Infectious Diseases and Tropical Medicine, Department of Medicine, Tri-Service General Hospital, National Defense Medical Center, Taipei 114, Taiwan; 4 Department of Medical Research, Taipei Tzu Chi Hospital, Buddhist Tzu Chi Medical Foundation, New Taipei City 231, Taiwan; kwtsai6733@gmail.com; 5 Department of Internal Medicine, Division of Nephrology, Tri-Service General Hospital, National Defense Medical Center, Taipei 114, Taiwan; wucc@mail.ndmctsgh.edu.tw; 6 Department and Graduate Institute of Microbiology and Immunology, National Defense Medical Center, Taipei 114, Taiwan; 7 Division of Nephrology, Department of Internal Medicine, Taipei Medical University Shuang Ho Hospital, New Taipei City 235, Taiwan; 11044@s.tmu.edu.tw; 8 Department of Pediatrics, Taoyuan Armed Forces General Hospital, Taoyuan City 325, Taiwan; lazyalways@gmail.com; 9 Division of Nephrology, Department of Medicine, Cardinal-Tien Hospital, School of Medicine, Fu-Jen Catholic University, New Taipei City 234, Taiwan; athletics910@gmail.com; 10 Division of Nephrology, Department of Medicine, Taipei Tzu Chi Hospital, Buddhist Tzu Chi Medical Foundation, New Taipei City 231, Taiwan; kuochenglu@gmail.com; 11 Division of Gastroenterology, Department of Internal Medicine, Taipei Tzu Chi Hospital, Buddhist Tzu Chi Medical Foundation, New Taipei City 231, Taiwan; chaoycmd@yahoo.com.tw

**Keywords:** adaptive immunity, COVID-19, innate immunity, vaccine, vitamin D

## Abstract

Severe acute respiratory syndrome coronavirus 2 is a new, highly pathogenic virus that has recently elicited a global pandemic called the 2019 coronavirus disease (COVID-19). COVID-19 is characterized by significant immune dysfunction, which is caused by strong but unregulated innate immunity with depressed adaptive immunity. Reduced and delayed responses to interferons (IFN-I/IFN-III) can increase the synthesis of proinflammatory cytokines and extensive immune cell infiltration into the airways, leading to pulmonary disease. The development of effective treatments for severe COVID-19 patients relies on our knowledge of the pathophysiological components of this imbalanced innate immune response. Strategies to address innate response factors will be essential. Significant efforts are currently underway to develop vaccines against SARS-CoV-2. COVID-19 vaccines, such as inactivated DNA, mRNA, and protein subunit vaccines, have already been applied in clinical use. Various vaccines display different levels of effectiveness, and it is important to continue to optimize and update their composition in order to increase their effectiveness. However, due to the continuous emergence of variant viruses, improving the immunity of the general public may also increase the effectiveness of the vaccines. Many observational studies have demonstrated that serum levels of vitamin D are inversely correlated with the incidence or severity of COVID-19. Extensive evidence has shown that vitamin D supplementation could be vital in mitigating the progression of COVID-19 to reduce its severity. Vitamin D defends against SARS-CoV-2 through a complex mechanism through interactions between the modulation of innate and adaptive immune reactions, ACE2 expression, and inhibition of the renin-angiotensin system (RAS). However, it remains unclear whether Vit-D also plays an important role in the effectiveness of different COVID-19 vaccines. Based on analysis of the molecular mechanism involved, we speculated that vit-D, via various immune signaling pathways, plays a complementary role in the development of vaccine efficacy.

## 1. Introduction

The 2019 coronavirus disease (COVID-19) poses a serious public health threat [1]. The pathogen, severe acute respiratory syndrome coronavirus 2 (SARS-CoV-2), belongs to the Betacoronavirus family. It usually causes respiratory symptoms [2]. Many studies have been conducted, and many strategies have been developed to prevent the spread of COVID-19 and to develop effective drugs and vaccines [3]. The structures of viral proteins, including the main protease (Mpro), spike protein (S protein), and RNA-dependent RNA polymerase (RdRp), have been elucidated [4,5], providing essential information for the manufacture of drugs against SARS-CoV-2. The realization of host immunity induced by SARS-CoV-2 has also sped up the development of vaccines and therapies. Multiple drugs and vaccines are under development to treat COVID-19. Some effective strategies have been developed to improve vaccine safety and efficacy [6].

A recent article regarding the effectiveness of two inactivated SARS-CoV-2 vaccines on cases of COVID-19 reported that the vaccine efficacy was around 72–78% in the United Arab Emirates and Bahrain [7]. In contrast, BNT162b2 and mRNA-1273 (both coding for the spike S1 protein) are two newly approved COVID-19 mRNA vaccines that have demonstrated excellent safety and effectiveness. BNT162b2 and mRNA-1273 have shown satisfactory safety and efficacy profiles, with an effectiveness of around 94–95%, based on data from the U.S. or mainly from the U.S. [8], where vitamin D food fortification has been mandatory for several years. Thus, we speculated that the relatively low vaccine efficacy of inactivated SARS-CoV-2 vaccines is due, at least in part, to low vitamin D levels in the study population (in the Middle East region). Whether vitamin D supplementation in the vitamin D deficiency population will mitigate this disadvantage merits further investigation.

This narrative review addresses the immune mechanism of the disease caused by SARS-Cov-2. Concurrently, we discuss the possible effect of vitamin D on the immune process of COVID-19. Then, we explore the immune protection mechanisms provided by various types of vaccines. We also analyze the potential benefits of vitamin D for a variety of vaccine formulations.

## 2. Immune Pathogenesis of COVID-19—Innate Immunity

### 2.1. Innate Immunity to SARS-CoV-2

**Figure 1:**
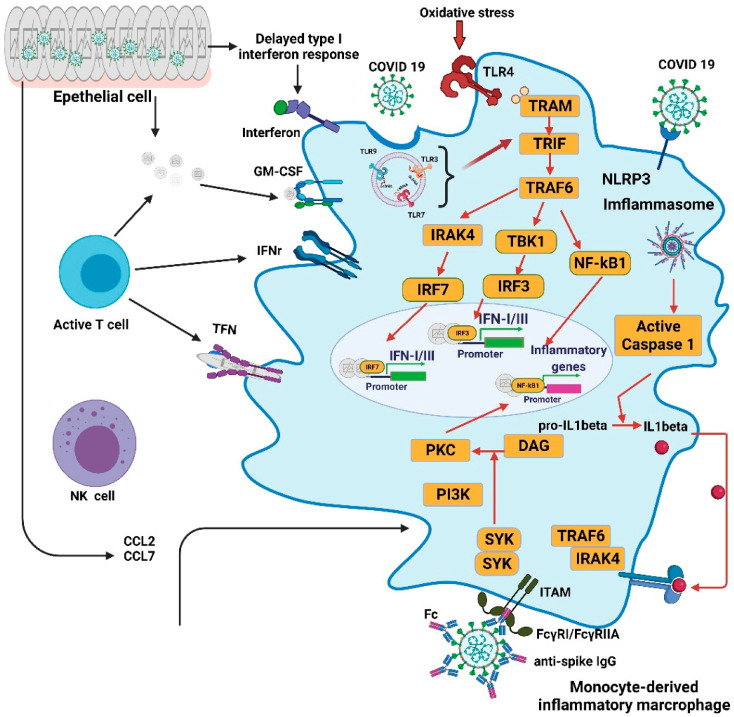
Overactivation of monocyte-derived macrophages and hyperactivity of inflammation in COVID-19. Several mechanisms can cause the overactivation of macrophages seen in patients with COVID-19. A delayed response by type I interferons leads to enhanced cytopathic effects of microbial threats and promotes the enhanced release of monocyte chemokines by alveolar epithelial cells, resulting in the recruitment of blood monocytes in the lungs. Monocytes differentiate into TH17-like proinflammatory macrophages through the activation of the Janus kinase (JAK)-signal transducer and activator of transcription 3 (STAT3) pathways. Proinflammatory cytokines, including granulocyte-macrophage colony-stimulating factor (GM-CSF), tumor necrosis factor-α (TNFα), and interferon-γ (IFNγ), further promote the recruitment and activation of monocyte-derived macrophages. Oxidized phospholipids (OxPLs) accumulate in infected lung epithelial cells and activate monocyte-derived macrophages through the Toll-like receptor 4 (TLR4)–TRAF6–NFκB pathway. IgG1 is the host antiviral neutralizing antibody, and RNA viruses usually mislead the infected host to produce non-neutralizing IgG3 antibodies. The binding virus and the IgG3 antibody can be taken up by the Fcγ receptor (CD64 or CD32) of macrophages. This process is called antibody-dependent enhancement (ADE). This enables the virus to gain access in order to infect macrophages and to activate the NLRP3 inflammasome, causing the secretion of mature IL-1β and IL-18. Interleukin-1β can enhance the activation of macrophages in an autocrine or paracrine manner, and it also reduces type I interferon production. Activated monocyte-derived macrophages contribute to the COVID-19 cytokine storm by releasing numerous proinflammatory cytokines. CCL, CC-chemokine ligand; CXCL10, CXC-chemokine ligand 10; ISG, interferon-stimulated gene; ITAM, immunoreceptor tyrosine-based activation motif; TRAM, TRIF-related adaptor molecule.

It is well known that COVID-19 can cause serious illness, which is characterized by significant immune dysfunction, stimulated by a vigorous but dysregulated innate immune response, along with a worse adaptive response, as shown in Figure 1 [9]. In SARS-CoV infection, the delayed response of type I interferon (IFN-I) results in the rapid replication of the virus, an abnormal increase in cytokines, and an abnormal response to chemokines, resulting in high mortality. The severity of the disease can be lessened by means of treatment with type 1 interferon before reaching the peak virus replication stage or by attenuating activated macrophages [10]. In patients with SARS, a prolonged response to IFN has been shown to delay the adaptive immune reaction. The continuous upregulation of inflammatory cytokines and the promotion of IFN-stimulated gene (ISG) expression illustrate the need to address IFN production in order to initiate protective adaptive immunity [11]. In other words, the unregulated IFN response during the acute phase of SARS-Cov-2 may eventually lead to the failure of the transition from innate immunity to adaptive immunity. The current data suggest that patients with severe COVID-19 may have postponed induction or no induction of IFN-I and -III [12]. In the absence of IFN-I or IFN-III, SARS-CoV-2 may replicate with high titers, leading to an exaggerated inflammatory response [9].

### 2.2. Interferon (IFN) Antagonism by SARS-CoV-2 Proteins

After virus infection in the respiratory tract, a signal cascade is induced, leading to the production of IFN, which acts in an autocrine and a paracrine manner to activate the IFN signaling pathway. In all immune cells, plasmacytoid dendritic cells (pDCs) have the particular role of secreting IFN-I during viral infection [13]. Pattern recognition receptors (PRRs) and inflammasomes are both key components of the innate immune system against viral pathogens, as they recognize conserved molecular motifs associated with viral pathogen-associated molecular patterns (PAMPs). The host’s immune response to viral infection depends critically on the initial activation of PRRs by the virus, primarily by viral DNA and RNA [14]. Receptor interacting protein kinase 1 (RIG-I)-like receptors (RLRs) belong to a family of cytoplasmic RNA receptors that play a critical role in detecting viral RNA genomes and replicative RNA intermediates. This receptor family includes RIG-I, melanoma differentiation-associated protein 5 (MDA5), and laboratory of genetics and physiology 2 (LGP2). The cytoplasmic receptor MDA5 is considered to be the most important factor in the detection of CoV RNA [15]. Activated PRRs activate downstream adaptors [16]. Receptor interacting protein kinase 1 (RIG-1) and melanoma differentiation-associated protein 5 (MDA5) signaling occur through the mitochondrial-anchored adaptor mitochondrial antiviral-signaling protein (MAVS), which signals through TBK1 and IKKє to initiate the NFκB and IRF family, leading to the expression of proinflammatory cytokines and type I interferons [17]. These adaptors trigger IRF3 and 7 (interferon regulatory factors) and promote their nuclear translocation to bind to DNA IFN promoters. Both IFN-I and IFN-III act on their own receptors and trigger the JAK/STAT signaling cascade to bind to the ISG 3 factor complex (ISGF3) with STAT1 and STAT2, resulting in the production of overall ISG. Type I IFNs (IFN-α/IFN-β) and type III IFNs (IFN-λ) share many characteristics, including the activation of shared signaling pathways, induction by viral infection, and later the transcriptional procedure [16]. The IFN-II signal leads to the formation of STAT1 homodimers and the production of numerous ISGs that partially overlap with STAT2. These signaling cascades are essential to inducing an antiviral status when viral infections occur [9].

SARS-CoV-2 genomes code four main structural proteins, which include the membrane (M), envelope (E), nucleocapsid (N), spike (S), and more than 16 nonstructural proteins (Nsps) that are all essential for viral survival. SARS-CoV-2 replication/transcription occurs in double-membrane vesicles, which shield the viral RNA from recognition by pattern recognition receptors [18]. There is also a varying number of auxiliary proteins, which are inserted between the structural genes and are needed to replicate the virus, but that contribute to avoiding host immunity and pathogenesis. Many of these viral genes counteract or prevent signaling to interferons, which can present in the form of an observed delay in IFN expression. These proteins not only act upstream of IFN production by weakening or antagonizing PRR recognition and signal transduction but also by directly weakening the IFN signaling pathway downstream, thereby promoting the early rapid replication of the virus. Therefore, SARS-CoV-2 has developed many routes to block the adequate IFN response [9].

### 2.3. Inflammatory Cytokine Response to SARS-CoV-2

Once the PRR detects viral RNA, IFNs and many other cytokines/chemokines are produced, which activate the signaling cascade of NF-kB, ultimately leading to the further expression of various cytokines/chemokines. This also activates the NOD-like receptor pyrin domain containing 3 (NLRP3) inflammasomes and facilitates the activation of proinflammatory cytokines [19]. Severe COVID-19 is disclosed by a significant increase in proinflammatory cytokines and a decrease in T lymphocytes [20]. However, the exact magnitude of inflammatory markers is not easy to establish because of inter-patients variability; however, a phenotype of elevated IL-6, IL-8, IL-10, and TNFa, accompanied by an assortment of chemokines, has been demonstrated [20,21,22].

When comparing sepsis with COVID-19 patients, serum IL-6 rates are typically five to 100 times higher in sepsis conditions than in patients with COVID-19 [23]. Therefore, it is not appropriate to characterize COVID-19 simply as a cytokine storm, and significant inflammatory dysregulation can be a more specific feature of the immune response [9]. A recent clinical study revealed that severe adaptive immunity suppression characterizes COVID-19 infections [24]. For this reason, patients with COVID-19 are generally not able to successfully induce protective antibodies to SARS-CoV-2.

### 2.4. Innate Cellular Immune Reaction to SARS-CoV-2

SARS-CoV-2 infection reveals the significant recruitment of immune monocytes/macrophages/neutrophils in the lung parenchyma. Pulmonary pathology indicates upregulated neutrophil and monocyte chemoattractants [25,26]. Pathological elucidation of infiltrating monocytes in the bronchoalveolar lavage (BAL) revealed the presence of vast monocytes with an inflammatory phenotype [27]. Proteomic analysis of the innate immune response pattern revealed that symptoms are rapidly followed by an influx of CD169+ monocytes and an abundance of IFN-γ+MCP-2+ expression. In the later stages of severe COVID-19, a persistent inflammatory phenotype with high levels of CCL3 and CCL4 is obtained. These are correlated with the re-emergence of CD16^+^ monocytes [28]. This kind of monocyte is a superior source of IL-6 and other dissimilar cytokines. Neutrophils have the ability to produce reactive oxygen species (SAR), and under oxidative stress, they may form extracellular neutrophil traps (NETs). These NETs are found in pulmonary and tracheal aspirations of patients with COVID-19 [29]. The production of ROS and NETs by neutrophils can aggravate the harmful response to infection and can aggravate the death of respiratory epithelial cells [29,30,31].

## 3. Adaptive Immune Reactions in SARS-CoV-2

### 3.1. Autoantibodies and B-Cell Reactions in COVID-19 Patients

Autoantibodies to interferons have been detected in patients with serious pneumonia caused by COVID-19. IgG autoantibodies against interferon-ω and interferon-α2 were detected. Therefore, these autoantibodies can neutralize interferon-ω and interferon-α2 in vitro. These data confirm the protective effect of interferons on the pathogenesis of SARS-CoV-2 in certain patients [32,33]. The exact mechanism by which B cells combat SARS-CoV-2 in vivo is not currently clear. Based on the ability of professional dendritic cells (DCs) and helper T cells to coordinate the response of B cells, it can be seen that viral antigens that enter lymph nodes by means of migrating APCs can cross-link B-cell receptors (BCRs) to activate antibody-mediated responses and memory B-cell development [34]. These observations indicate that DCs play an important role in initiating Ab synthesis through direct interaction with B cells. For the B-cell response, the help of CD4 T cells is essential. The CD4 T helper cell response after SARS-CoV-2 infection is related to protective IgG and IgA antibody levels, ensuring the T-cell-dependent B-cell response after COVID-19 illness [35]. This also suggests the reactive cross-recognition of T cells between circulating “cold” coronaviruses and SARS-CoV-2. The CD4 T helper cell response in SARS-CoV-2 appears to involve THαβ, which provides the main host immunity against viruses [36]. THαβ cells have been called Tr1 cells in previous studies. THαβ immunity is driven by IFN-I or IL-10. The major effector cells of THαβ immunity are NK cells, IL-10-producing CD4 T helper cells, CD8 T cytotoxic cells, and IgG1 B cells. The key transcription factors are STAT1, STAT2, and STAT3β. THαβ immunity is related to type 2 antibody-dependent cytotoxic hypersensitivity, such as that observed in myasthenia gravis. IL-10 is not a purely immunosuppressive cytokine; it can have potent stimulatory effects on NK cells, CTLs, and B cells. The CD4 T cells and CD8 T cells reacting to SARS-CoV-2 infection appear to be effector T lymphocytes, which are required to generate robust memory T and B immunities against reinfections.

By studying antibody, memory B cell, memory CD4+ T cell, and memory CD8+ T-cell responses to SARS-CoV-2 in an integrated manner [35,37]. Dan et al. showed that memory B lymphocytes and memory CD4 and CD8 lymphocytes are maintained for 8 months following infection with SARS-CoV-2. The SARS-CoV-2 spike protein RBD-specific anti-IgG titer is related to neutralizing effects [38], and anti-IgA antibodies can be detected from six to eight months after infection. Similarly, memory B lymphocytes expressing the IgG1 isotype show the same persistence pattern. SARS-CoV-2-specific memory CD4 T helper and CD8 T cytotoxic cells were also detected, but their number decreased by about 50% in 6–8 months, compared with memory B cells [37]. Another study also showed that RBD-specific IgG and IgM decreased significantly after SARS-CoV-2 infection, but the neutralizing antibody decreased by only ~50% in infected patients after 6 months [39]. Interestingly, the IgG antibody of convalescent rhesus macaques infected with SARS-CoV-2 protected naive rhesus macaques from a SARS-CoV-2 challenge [40]. These studies demonstrated that both B and T lymphocytes mediated effector and memory immunities against SARS-CoV-2 that can persist and provide long-term immune reactions against reinfections.

### 3.2. Crosstalk between Innate and Adaptive Immunity with SARS-CoV-2 Infection

The mechanism by which antigen-specific T cells differentiate into memory or effector immune cells after SARS-CoV-2 infection is unknown. In acute viral infections, naive CD8 T lymphocytes are activated following interactions with professional antigen-presenting cells. Activated T cells act and eradicate virus-infected cells [41,42]. With the eradication of the acute infection, most antigen-reactive T lymphocytes are programmed into terminal differentiation and apoptosis processes, and only a small percentage of lymphocytes survive and divide into memory T-cell lineages. CD4 helper T cells secrete cytokines and are critical for B-cell survival, antibody isotype class change and reorganization, and CD8 memory T-cell production [43]. The interaction of the innate immune system with the adaptive immune reaction after SARS-CoV-2 infection has recently been studied. One major study by Grant et al. explored the crossover between innate tissue-resident alveolar macrophages (TRAMs) and adaptive T cells in patients with critical SARS-CoV-2 disease. In the majority of SARS-CoV-2 infected patients, the alveolar space was continually enriched with T lymphocytes and monocytes. These T lymphocytes are traditional TH1 CD4 T cells that produce interferon-γ to induce the release of cytokines from alveolar macrophages and further enhance the activation of T lymphocytes [44]. Additional results obtained through RNA sequencing studies conducted with peripheral leukocytes and bronchoalveolar lavage analyses demonstrated the occurrence of SARS-CoV-2-specific effector CD8 T cytotoxic lymphocyte reactions in patients with mild or moderately severe COVID-19. The effect of CD8 T cells appears to decrease in severely ill patients [45]. NK cells not only generate cytokines, such as IFN-II and interleukin-10 but also kill virus-infected cells. The total number of NK cells in the blood of COVID-19 patients is considerably smaller than that of healthy controls [46]. NK cells are present with elevated levels of granzyme B and perforin, which are involved in antibody-dependent cell cytotoxicity. This evidence suggests that cytokine-producing NK cells could be implicated in host immunity in patients with COVID-19 [9].

## 4. Vitamin D and Immunity

### 4.1. Antiviral Activity of Vitamin D and the Innate Immune Response

Patients with respiratory diseases often present with a lack of vitamin D; vitamin D supplementation could provide substantial benefits to the above population [47,48]. After binding to serum vitamin D binding protein, circulating 25-hydroxyvitamin D enters monocytes and increases the intracellular level of active 1,25-dihydroxyvitamin D (1,25D). After binding to vitamin D receptor (VDR), 1,25D induces antimicrobial peptides cathelicidin and β-defensin 4A and promotes autophagy through autophagosome formation [49]. In humans, cathelicidin [49] and β-defensin [50,51] are produced through a vitamin D-dependent antimicrobial pathway. Our previous studies also demonstrated that vitamin D-treated uremic hyperparathyroidism can efficiently increase serum cathelicidin levels [52]. Vitamin D’s promotion of antiviral immunity is closely related to COVID-19, involving various mechanisms that overlap with antibacterial responses, such as the induction of the expression of cathelicidin and defensins, which can also prevent viruses from entering cells and serve as an inhibitor of virus replication [53,54]. Another characteristic of vitamin D concerning antibacterial and antiviral mechanisms acts through the promotion of autophagy [55]. Autophagy exhibits dual effects during viral infections that promote the clearance of viral components and activate the immune system to produce antiviral cytokines. Specifically, autophagy encapsulation packs viral particles for lysosomal degradation and antigen presentation and the subsequent activation of adaptive antiviral immune responses [56]. Thus, autophagy facilitates a cellular environment that is hostile to viruses. Moreover, type I interferon (IFN-I) is a crucial antiviral factor, and studies have shown that autophagy affects IFN-I responses by regulating the expression of IFN-I and its receptors. Similarly, IFN-I and interferon-stimulated gene (ISG) products can mediate autophagy to promote antiviral immunity. Virus-induced autophagy can suppress IFN-I antiviral responses, but the IFN-I system can also manipulate autophagy to eliminate viruses. The crosstalk between autophagy and IFN-I responses can link autophagy to the antiviral immune response [56].

TLRs are transmembrane proteins that can recognize conserved molecular motifs derived from viruses and bacteria and trigger innate immune responses against these pathogens. TLR3 recognizes the double-strand RNA of the virus or synthetic double-strand RNA and is primarily involved in the defense of the virus. Vitamin D therapy has been shown to reduce the expression of chemokines in respiratory epithelial cells through the RNA-TLR3 signaling pathway [57,58].

Together, vitamin D promotes innate immunity through the expression of cathelicidin and β-defensin, improves autophagy, accelerates and cooperates with IFN, and affects complement activation [59].

### 4.2. Vitamin D Regulates Adaptive Immunity 

The adaptive immune system is initiated by the antigen activation of antigen-presenting cells (such as dendritic cells and macrophages), which then activate antigen-recognizing cells, including T lymphocytes and B lymphocytes, which are the main determinants of the immune response [57]. 1α,25-dihydroxyvitamin D blocks NF-κB p65 activation by upregulating the NF-κB inhibitor protein IκBα and directly regulates inflammatory cytokines that depend on the activity of NF-κB in multiple cells (including macrophages) [60].

Circulating T cells, B cells, and dendritic cells express the vitamin D-activating enzyme CYP27B1 (1α-hydroxylase) and the VDR, which then use the circulating 25D through intracrine conversion to bioactive 1,25D. Increased intracellular 1,25D inhibits the maturation of dendritic cells and regulates the function of CD4^+^ T cells. In general, vitamin D modulates adaptive immunity by promoting the shift from TH1 to THαβ cells. In essence, vitamin D inhibits the activation of type 1 T helper cells and TH1 immune responses. Furthermore, vitamin D promotes the association of THαβ cells with anti-virus immunity, which improves the production of interleukin-10 and antiviral IgG1 from B-cell lineages [61]. Vitamin D also attenuates proinflammatory cytokine-related inflammation and tissue injury by inhibiting the development of Th17 cells. Likewise, Tregs suppress inflammation in response to vitamin D [62]. In brief, vitamin D is assumed to modulate adaptive immunity against COVID-19 in several ways. For example, it can suppress the maturation of dendritic cells and weaken the antigen presentation, and then increase cytokine production induced by CD4^+^ T cells and promote the effectiveness of Treg lymphocytes. A recent clinical study revealed that COVID-19 infections are characterized by severe immunosuppression, especially of adaptive immunity, but not major cytokine storms [24]. Vitamin D may suppress TH1 and TH17 cytokine secretion and associated tissue destruction. It is assumed that these beneficial effects will occur even during COVID-19 infection, suggesting that appropriate vitamin D supplementation may reduce proinflammatory reactions and increase the anti-inflammatory effects of COVID-19.

### 4.3. Vitamin D Modulates ACE2 and the RAS 

Vitamin D deficiency is a global public health problem that varies with age, ethnicity, and latitude. The presence of comorbid diseases, such as septicemia, diabetes mellitus, chronic respiratory diseases, and malignancy, is tightly linked to vitamin D deficiency [63]. In the midst of the COVID-19 pandemic, a similarity in prevalent SARS-CoV-2 infection areas and vitamin D deficiency areas has been observed [64], which may show the importance of vitamin D supplementation in COVID-19 [65]. Adequate vitamin D levels are also required in order to reduce RAS activity and increase ACE2 concentrations in acute lung injury. Specifically, sufficient vitamin D supplementation can induce the ACE2/Ang 1–7 axis and inhibit the renin axis and the ACE/Ang II/AT1R axis [66].

The prognosis for COVID-19 among the elderly, smokers, and people with obesity or other comorbidities, including hypertension and diabetes mellitus(DM), is poor. RAS agents that increase ACE2 concentrations are used as a substrate for SARS-CoV-2 infection [67]. Circulating ACE2 is considered a biomarker of hypertension and heart failure [68] as well as DM [69]. Infection with SARS-CoV-2 decreases ACE2 activity and accumulates toxic Ang II and metabolites, which are then converted to ARDS or fulminant myocarditis [67]. Vitamin D sufficiency can lower RAS activity through several pathways, including transcriptional suppression of renin, ACE, and Ang II expression [70] and increased ACE2 concentration in lipoprotein (LPS)-induced acute lung injury (ALI) [66]. In other words, vitamin D attenuates LPS-induced ALI by inducing the ACE2/Ang 1–7 axis and inhibiting both renin and the ACE/Ang II/AT1R axis [66]. Vitamin D treatment also increases soluble ACE2 (sACE2) [71,72], which maintains the enzyme activity of ACE2 and may bind to the S protein of SARS-CoV. Thus, sACE2 can block the S protein and prevent cells from being infected.

ACE2 expression decreases in DM patients, possibly due to a high level of glucose-related glycosylation [73,74]; this could explain the increase in susceptibility to severe lung damage and ARDS associated with COVID-19. As a result, we can speculate on the beneficial effect of vitamin D supplementation on diabetic patients with COVID-19. In sum, vitamin D may be able to combat COVID-19 and the related induction of MAS and ARDS by targeting ACE2 downregulation and unbalanced RAS.

### 4.4. Vit-D Interplay with Antiviral IFN-I

**Figure 2:**
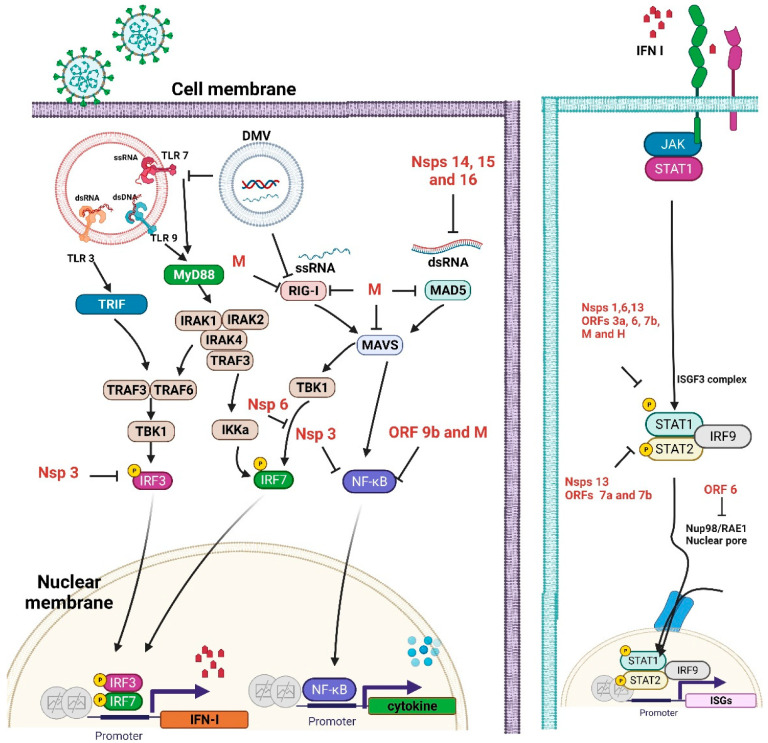
Innate immune system dampened by SARS-CoV-2 proteins.

Type I IFNs are the strongest natural antiviral mediators in humans [75], and there is overwhelming evidence that a weak or delayed response of Type I IFNs contributes to the severity of COVID-19 [76]. Vitamin D works directly against the hepatitis C virus (HCV). It enhances the IFN-α-mediated inhibition of HCV replication by inducing the induction of IFN-stimulated genes (ISGs) [77,78]. The combined therapy of infected human hepatocytes with low doses of IFN-α and vitamin D, which separately have weak antiviral effects, potently inhibited viral replication. This synergistic effect suggests that vitamin D potentiates IFN-α action [79]. Moreover, a molecular study [78] described a constitutive inhibitory interaction between vitamin D receptors (VDR) and STAT1. The release of STAT1 during stimulation with calcitriol suggests that unbound VDR could sequester STAT1, a key transcription factor in type I IFN signaling. Consequently, vitamin D deficiency could cause a less effective IFN-mediated antiviral reaction due to higher levels of unbound VDR. The damped type 1 interferon reaction by SARS-CoV-2 is shown in Figure 2.

Vitamin D was shown to exhibit antiviral activity against rhinoviruses [80] through increased virus-induced antiviral ISG expression. The study of peripheral blood cells in patients with multiple sclerosis (MS) revealed increased 25 (OH)D levels, resulting in reduced MS activity [78,81]. They discovered a complex network of 25(OH)D-regulated genes and verified known targets for IFN-β and other antiviral genes. Furthermore, both vitamin D [66] and type I interferon [82] may upregulate ACE2, which is a component of the renin-angiotensin system used by SARS-CoV-2 as a cell receptor. The effects of increased ACE2 expression can provide other protective effects in COVID-19.

According to the above evidence, we put forward the hypothesis that sufficient vitamin D status at the time of infection contributes to an early type I IFN protective response and enhances the innate antiviral immunity to SARS-CoV-2. As the disease progresses, the immunomodulatory activity of vitamin D may actually help to reduce the excessive inflammatory damage observed in severe COVID-19, proving its rationality as an adjuvant treatment [47,65,79].

Coronavirus replication occurs in double-membraned vesicles (DMVs). Its replication can shield viral ssRNA and viral dsRNA, recognized by PRRs such as TLR3, TLR7, RIG-I, and MDA5. These PRRs activate the adaptors TRIF and MyD88, which are downstream of TLRs, and MAVS and TBK1, which are downstream of RIG-I and MDA5. These steps are the initiation of the type 1 interferon production pathway. IRF3, IRF7, or NFκB activation results in the signaling. These proteins can activate downstream genes. Then, the immune gene transcription, including proinflammatory cytokines and interferons, is upregulated. The right-side solid line indicates the IFN signaling, beginning with IFN-I binding IFNAR to initiate JAK/STAT signaling and the formation of the ISGF3 complex STAT1/STAT2/IRF9, which activates ISRE transcription. SARS-CoV-2-encoded proteins (shown in red) inhibit many aspects of these pathways, resulting in decreased type 1 interferon and dysregulated proinflammatory cytokine expression. Many of the SARS-CoV-2 interferon antagonists have been identified in vitro and in vivo (black) [9]. The immune evasion displayed by SARS-CoV-2 includes pathogen sensing, interferon production, and ISG functions. Viral proteins can block one or multiple critical signaling molecules. In the beginning, viruses change their nucleic acid structures to avoid binding by host receptors. Viral RNA is guanosine-capped and 5′ end methylated by SARS-CoV nonstructural components (nsp10, nsp14, nsp15, and nsp16), allowing the CoV to avoid the binding of host dsRNA binders [83,84]. These are critical mechanisms by which SARS-CoV escapes from host immunity. Viral proteins suppress key molecules in the recognition of viral pathogens; for example, the SARS-CoV N protein and M protein can block RIG-I activation. In addition, other viral components suppress different signaling cascades for the induction of interferons; SARS-CoV-2 ORF9b interacts with MAVS in mitochondria. The endoplasmic reticulum STING signaling is stopped by the SARS-CoV protein nsp3. SARS-CoV employs additional signaling interruption mechanisms. SARS-CoV-2 nsp13 and nsp15 prevent TBK-1 and IRF3 activation. Viral proteins can suppress the function of transcriptional factors for the induction of IFNs or inflammatory cytokines. Furthermore, the SARS-CoV-2 nsp1 protein inhibits host gene expression by promoting the degradation of mRNA degradation and suppressing protein synthesis, including molecules included in host innate immune functions. The right panel shows how viral proteins block interferon signaling. Finally, SARS-CoV-2 Nsp3 is responsible for the inhibition of host innate immune responses through post-translational modification by ISG15.

## 5. Immunogenicity and Clinical Application of COVID-19 Vaccine

**Figure 3:**
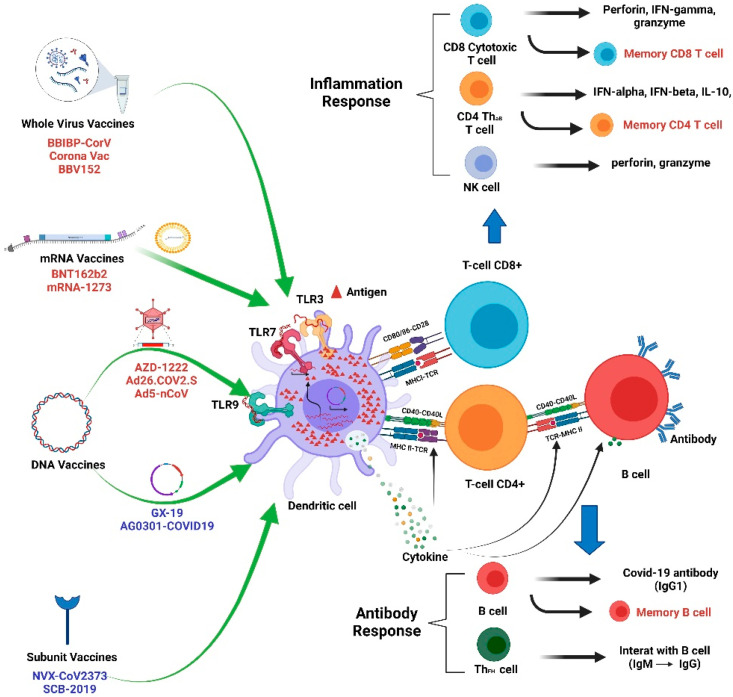
Immunogenicity of COVID-19 vaccines.

There are several available vaccines that have been developed for COVID-19. These consist of mRNA vaccines, DNA vaccines, protein subunit vaccines, and inactivated vaccines. These vaccines aim to induce antiviral immune responses. Some of these vaccines show satisfactory efficacy against SARS-CoV-2. We discuss these vaccines below. The summary of these vaccine mechanisms is shown in Figure 3.

Inactivated SARS-CoV-2 vaccines produce neutralizing antibodies to the live SARS-CoV-2 antigen, IgG antibodies specific to the whole SARS-CoV-2 antigen [85], SARS-CoV-2 IgG titers against the spike protein, receptor-binding domain (RBD), and nucleocapsid IgG; increase the anti-spike protein IgG1/IgG4 ratio; and elicit IFN-γ-positive CD3^+^, CD4^+^, and CD8^+^ T-cell proliferation [86]. The mRNA vaccine can produce specific RBD antibody titers and neutralizing antibody concentrations that are significantly higher than those seen in people recovering from COVID-19. In addition, the vaccine-induced T-cell response is oriented toward a TH1 response, and no evidence of vaccine-enhanced illness has been reported [8]. All these immunogenicity processes are related to vitamin D status.

COVID-19 vaccine-induced host immune reactions. Whole-virus vaccines (BBIBP-CoV, Corona Vac, and BBV152) activate TLR3, TLR7, and TLR9 to trigger an antiviral THαβ immune response. mRNA vaccines (BNT162b2 and mRNA-1273) activate TLR3 and TLR7 to trigger an antiviral THαβ immune response. DNA vaccines (AZD-1222, Ad26.COV2.S, AdSnCoV, GX19, and AG0301-COVID19) activate TLR9 and later TLR3/TLR7 to trigger THαβ immunity. Subunit vaccines (NVX-CoV2373 and SCB-2019) activate TLR3, TLR7, and TLR9 with the help of adjuvants to trigger an antiviral THαβ immune response. THαβ immunity includes IFN-I- and IL-10-secreting CD4 T cells, NK cells, CD8 T cells, and IgG1 B cells. The follicular helper T cells (ThFH) can help in B-cell antibody isotype switching from IgM to IgG. NK cells and CD8 T cells can mediate ADCC and viral infected cell apoptosis via granzymes and perforins. The vaccines can induce the activation of long-term memory B cells, memory CD4 T cells, and memory CD8 T cells.

## 6. Gene-Based Vaccines

### 6.1. mRNA COVID-19 Vaccines and Immunity

#### 6.1.1. Mechanisms of Immunogenicity

The mechanism of mRNA vaccines is discussed below. mRNA vaccines, including the BNT162 vaccine and the Moderna vaccine, use modified mRNA to initiate the human host’s immune reaction. mRNA vaccines encode the sequence of the SARS-CoV2 spike protein. Once the mRNA vaccine is injected into the body, macrophages or dendritic cells can uptake the mRNA fragment because it represents foreign content in the body. The mRNA fragment is taken by macrophages or dendritic cells via phagocytosis. RNA contents are most sensitive to being taken up by plasmacytoid dendritic cells (pDC). Macrophages or plasmacytoid dendritic cells then migrate to the nearby lymph nodes via lymph circulation. These macrophages or plasmacytoid dendritic cells enter lymph nodes and transmit the antigens to lymph node-resident follicular dendritic cells (FDC). Follicular dendritic cells then secrete CXCL13 chemokines to accumulate CXCR5 (CXCL13 receptor)-bearing follicular helper T cells with the help of lymphoid tissue inducer (LTi) cells through the action of secreted lymphotoxin. Follicular helper T cells present antigens to B cells in germinal centers to promote antibody switching from IgM to IgG [6].

In addition, IL-10-producing innate lymphoid cells (ILC10) help to secret interleukin-10. Once the mRNA antigen is taken up by plasmacytoid dendritic cells, it binds to Toll-like receptor 3 and Toll-like receptor 7. These toll-like receptors bind to single- or double-stranded RNA molecules. Then, IRF3 and IRF7 signaling is triggered, upregulating type 1 interferons. Plasmacytoid dendritic cells then secrete an amount of type 1 interferons (IFNα, IFNβ). mRNA can translate into spike protein antigens. Plasmacytoid dendritic cells also present antigens to T helper cells. The combined effects of interleukin-10 and type 1 interferon trigger T helper cells to become THαβ (Tr1) helper T cells. STAT1, STAT2, and STAT3β are upregulated, triggering the antiviral immunological pathway. Tr1 cells can produce large amounts of interleukin-10, promoting the B-cell antibody to become antiviral IgG1. Plasmacytoid dendritic cells also cross-present antigens to cytotoxic T cells to activate them, with specific TCR reacting to the SARS-CoV2 spike protein. These cytotoxic T cells can directly kill virus-infected cells.

#### 6.1.2. Clinical Immunological and Vaccine Efficacy Profiles

The Pfizer BNT162b2 vaccine is an mRNA-based vaccine. Clinical trials were conducted in adults in the United States and Germany [87]. Both the phase I and phase II trials showed the safety and immunogenicity of the BNT162b2 mRNA vaccine [88]. Both BNT162b1 and BNT162b2 mRNA vaccines were tested in the clinical trials, but the results showed the advantage of the BNT162b2 mRNA vaccine. A dose-response relationship was found in these clinical trials. The phase 1 clinical trials were conducted in the USA and Germany. Healthy adults aged from 18 to 55 years old and older adults aged from 65 to 85 years old received either placebos or one of two BNT mRNA vaccines. The BNT162b1 vaccine encodes a secreted trimerized SARS-CoV-2 receptor-binding domain (RBD), whereas the BNT162b2 vaccine encodes a whole SARS-CoV-2 spike RNA sequence. The primary outcome was safety (local or systemic adverse effects), and the secondary outcome was immunogenicity. To test the dose-response relationship, vaccine dosages of 10, 20, 30, and 100 μg were given in the trial group. In one study group of the clinical trial, they received two doses of the mRNA vaccine with a 21-day interval.

In the results, the BNT162b2 vaccine presented a lower incidence or severity of adverse effects in study populations compared to those of the BNT162b1 vaccine. Its safety was apparent in older adults. In both BNT162b1 and BNT162b2 vaccine groups, younger and older study populations both elicited significant SARS-CoV-2-neutralizing antibodies, so the immunogenicity of both vaccines was similarly excellent. However, the BNT162b2 vaccine was chosen for further usage due to safety and tolerability issues.

The Moderna mRNA-1273 vaccine is another mRNA-based vaccine developed in the USA [89]. The phase 3 randomized controlled trial was conducted in the United States. People at higher risk for SARS-CoV-2 infection were randomly assigned to receive two injections of the mRNA-1273 vaccine or a placebo 28 days apart. The primary endpoint was the prevention of COVID-19 disease in those who had not previously been infected with SARS-CoV-2. In the results, symptomatic COVID-19 illness was noted in 185 participants in the placebo group and in 11 participants in the mRNA-1273 group; the vaccine efficacy was 94.1%. Mild to moderate adverse reactions after vaccination occurred more frequently in the mRNA-1273 vaccine group. Serious adverse effects were very rare, and the incidence was the same in the two groups. Thus, the mRNA-1273 vaccine obtained 94.1% efficacy in preventing COVID-19, including protecting against severe disease.

### 6.2. DNA COVID-19 Vaccine and Immunity

#### 6.2.1. Mechanisms of Immunogenicity

DNA vaccines such as the AstraZeneca Oxford vaccine and the Johnson and Johnson vaccine use adenoviral vectors to incorporate the SARS-CoV-2 spike protein gene sequence in this viral vector [90]. The DNA is injected into the body to generate immunity against SARS-CoV2. The DNA molecule injected into the body can be taken up by plasmacytoid dendritic cells and macrophages. These antigen-presenting cells then present the antigen to CD4 T cells to trigger adaptive immunity. The difference between DNA vaccines and RNA vaccines is that DNA uses Toll-like receptor 9 (TLR9) as the cellular receptor to generate cellular signaling to trigger IRF7 to activate type 1 interferons, including IFNα and IFNβ. When DNA is transcribed into RNA, Toll-like receptor 3 and Toll-like receptor 7 are also used to recognize double- or single-stranded RNA molecules. The process is similar to that of mRNA vaccines in the generation of antiviral immunity. To trigger successful host immunity, the dendritic cells first migrate to nearby lymph nodes. With the incorporation of follicular dendritic cells and lymphoid tissue inducer cells, follicular helper T cells allow germinal center B-cell isotype switching from IgM to IgG to generate memory B cells and effector B cells.

For the triggering of THαβ immunity, plasmacytoid dendritic cells are still the most important antigen-presenting cells. With the help of IL-10-producing innate lymphoid cells 10 secreting type 1 interferon and interleukin 10, the antigen-presenting cells present viral antigens to CD4 T cells. Thus, CD4 T cells become THαβ CD4 T cells, producing large amounts of interleukin 10. Via the cross-presentation process, CD8 T cells are also activated to recognize viral antigens. Interleukin 10 also causes B-cell isotype switching to anti-virus IgG1 antibodies. Interleukin 10 can also activate NK cells and CD8 T cells. Through the above mechanisms, memory T cells and memory B cells are generated for long-term immunity.

#### 6.2.2. Clinical Immunological and Vaccine Efficacy Profiles

The efficacy of the typical AstraZeneca DNA vaccine is discussed below. Phase 2 and phase 3 trials have been conducted to test the vaccine’s safety and efficacy in the United Kingdom [91]. The participants’ ages were stratified into 18–55 years old, 56–69 years old, and over 70 years old. The results showed that local and systemic effects (local pain, fever, muscle pain, and headache) were noted more in the vaccine group than in the placebo group. However, no lethal adverse effects were found. The above side effects were seen more in the younger participants (aged <56 years old). In participants who received two full doses of the vaccine, the anti-spike SARS-CoV-2 IgG reactions 28 days after the second booster dose were the same across the three age groups. These three groups generated satisfactory anti-SARS-CoV-2 neutralizing antibodies. Neutralizing antibodies after a second boost dose were similar across all age groups. T-cell responses peaked at day 14 after the first standard dose of the AstraZeneca vaccine. The vaccine’s efficacy was satisfactory.

## 7. Inactivated and Protein Subunit COVID-19 Vaccines and Immunity

### 7.1. Inactivated COVID-19 Vaccines and Immunity

#### 7.1.1. Mechanisms of Immunogenicity

Inactivated COVID-19 vaccines, including the Sinovax vaccine, can trigger host immunity against the virus in several aspects. Inactivated COVID-19 is treated using an inactivating agent such as formaldehyde to allow SARS-CoV2 to become non-infectious. Thus, the components of inactivated COVID-19 vaccines are complicated. The contents are RNA and proteins, including the nucleocapsid protein and spike protein. All the components have the potential to initiate a host immune response. Once the inactivated vaccine is injected into the body, macrophages and dendritic cells digest these foreign antigens. Plasmacytoid dendritic cells take up the RNA components. Myeloid dendritic cells and macrophages intake the protein components, including the nucleocapsid protein and spike protein. In plasmacytoid dendritic cells, RNA binds to Toll-like receptor 3, Toll-like receptor 7, and Toll-like receptor 9 to initiate the response of type 1 interferons. In myeloid dendritic cells or macrophages, the uptake of protein antigens can trigger Toll-like receptor 7, Toll-like receptor 8, and Toll-like receptor 9. These can trigger TH1 or THαβ immune responses. In addition, dendritic cells also migrate to lymph nodes to activate follicular helper T cells and subsequent B-cell isotype switching from IgM to IgG1. CD8 T cells are also activated by these antigen-presenting cells [6].

#### 7.1.2. Clinical Immunological and Vaccine Efficacy Profiles

The Sinovac vaccine is an inactivated vaccine developed in China. Clinical trials to test its efficacy and tolerability have been performed in the Jiangsu Province of China [92]. In this randomized controlled trial, healthy adults aged from 18 to 59 years old were recruited. In the phase 1 trials, the incidence of adverse effects for day 0 and day 14 cohorts was 29% in the 3 ug group, 38% in the 6 μg group, and 8% in the placebo group. Neutralizing antibodies on day 14 after day 0 and day 14 in the vaccination schedule were seen in 46% of 24 participants in the 3 μg group, 50% in the 6 μg group, and 0% in the placebo group. On day 0 and day 28, neutralizing antibodies were seen in 83% of participants in the 3 μg group, 79% of participants in the 6 μg group, and 4% of participants in the placebo group. In the phase 2 trial, the incidence of adverse effects for participants on day 0 and day 14 was 33% in the 3 μg group, 35% in the 6 μg group, and 22% in the placebo group. Neutralizing antibodies were noted for 92% of participants in the 3 μg group, 98% in the 6 μg group, and 3% in the placebo group at day 14 after the zero-day and 14-day schedules. At day 28 after the zero-day and 28-day schedules, neutralizing antibodies were seen in 97% in the 3 μg group, 100% in the 6 μg group, and 0% in the placebo group. The overall efficacy and tolerability of the vaccine were satisfactory.

### 7.2. Protein Subunit COVID-19 Vaccines and Immunity

#### 7.2.1. Mechanisms of Immunogenicity

Subunit vaccines, such as the Novavax vaccine, use protein antigens to trigger a host immune response. The mechanism of the subunit vaccine is similar to that of the HBV vaccine and the HBsAg subunit vaccine. No RNA content is used in subunit COVID-19 vaccines. Once the subunit vaccine is injected into the body, macrophages and dendritic cells take in these protein antigens via phagocytosis. Then, the protein antigen binds to Toll-like receptor 7, Toll-like receptor 8, and Toll-like receptor 9 to trigger a type 1 interferon response to initiate a TH1 or THαβ immune reaction in the myeloid dendritic cells. In addition, dendritic cells also migrate to lymph nodes to activate follicular helper T cells and subsequent B-cell isotype switching from IgM to IgG1. CD8 T cells are also activated by these antigen-presenting cells. Because of the lack of RNA content in the protein subunit vaccines, they may not trigger optimal antiviral immunity successfully. Another issue of subunit vaccines is that they may not be very successful against SARS-CoV2 variants, which cause mutations of the spike protein. In addition, the protein antigen may not fully initiate antiviral THαβ immunity. It may also induce TH1 immunity against intracellular bacteria or protozoa. However, the advantage of subunit protein vaccines is their relative safety, with fewer adverse effects after vaccination [6].

#### 7.2.2. Clinical Immunological and Vaccine Efficacy Profiles

The Novavax COVID-19 vaccine is made from the full-length spike (S) protein. Matrix-M adjuvants significantly increased the vaccine’s immunogenicity, resulting in antigen-specific humoral and cellular immune responses, activating follicular helper T cells (Tfh) and antigen-specific germinal center (GC) B cells in lymph nodes. In baboons, low-dose levels of NVX-CoV2373 with Matrix-M elicited high titers of anti-S antibodies that block S-protein binding to hACE2 and neutralize virus infection by initiating antigen-specific T cells, including CD4 T cells and CD8 T cells.

The efficacy of the Novavax vaccine (NVX-CoV2373) was assessed in a phase 2a/b clinical trial conducted in South Africa [93]. Both HIV-positive and -negative adults aged 18 to 64 years old were recruited. The NVX-CoV2373 vaccine is made from the recombinant spike protein with Matrix-M1 adjuvants. In the results of the trial, the vaccine’s efficacy among HIV-negative participants was 60.1%. This efficacy was higher in HIV-negative participants than in HIV-positive participants. Local and systemic side effects were more common in the vaccine group; severe reactions were very rare in both groups. Thus, the NVX-CoV2373 vaccine was efficacious in preventing COVID-19. Higher vaccine efficacy was observed among HIV-negative participants.

## 8. Possible Links between Vitamin D and Vaccine Effectiveness

At present, significant efforts have been made to develop effective and safe vaccines for SARS-CoV-2, resulting in the development of inactivated vaccines, DNA/mRNA vaccines, and protein subunit vaccines [6]. However, the role of vitamin D in the effectiveness of these vaccines has not yet been confirmed by further studies.

Vitamin D deficiency (VDD) occurs all over the world, mainly in the Middle East, China, Mongolia, and India [94]. The question of whether VDD affects immune responses to influenza immunization is inconclusive. Seroprotection (SP) rates of subtype H3N2 (A/H3N2) and strain B of influenza A virus in VDD patients are lower than those of patients with normal levels of vitamin D [95]. Vitamin D deficiency is prevalent among COVID-19 patients. A study based on the Israeli population showed that a low level of vitamin D in plasma of 25(OH) is associated with a higher risk of COVID-19 infection [96]. Low levels of 25(OH)D at hospitalization have been associated with the COVID-19 stage and mortality [97]. Correlations have been observed between the historical prevalence of vitamin D deficiency and COVID-19 mortality in European countries [98]. The amazingly high levels of vitamin D in Scandinavian countries reflect their policy of vitamin D fortification and supplementation [99]. Systematic vitamin D food fortification is an effective approach to improve vitamin D deficiency in the general population and has already been introduced by countries such as the U.S., Canada, and Finland [100].

Currently, dark skin color, age, pre-existing conditions, and vitamin D deficiency are characteristics of patients with severe COVID-19. Among these, only vitamin D deficiency can be modified. Numerous observational studies have provided evidence that serum 25-hydroxyvitamin D levels are inversely correlated with the incidence and severity of COVID-19. These observations support our hypotheses. This evidence to date generally satisfies Hill’s criteria for causality in a biological system, such as strength of association, consistency, temporality, biological gradient, plausibility, and coherence, although experimental verification is lacking [101].

Experience in the development of SARS-CoV vaccines has raised concerns about the correlation between pulmonary histopathology and immune responses to Th2 cytokines [102]. Th2 cells can secrete many cytokines, such as IL-4, IL-5, IL-10, and IL-13. Aberrantly high levels of Th2 cytokines can elicit immune responses that prompt eosinophilic infiltrations. Four different SARS-CoV vaccines led to the development of Th2-type immunopathology with elevated eosinophilic infiltration, which represented a Th2-type hypersensitivity marker in mouse models [103]. Similar results were observed in inactivated MERS-CoV vaccines that also showed eosinophilic infiltration, with IL-5 and IL-13 levels higher than those that existed before vaccination in mouse models [104] content-type="background:white">. The immune response after vaccination can be partially attributed to the presence of the nucleocapsid (N) protein in the vaccine [105]. Studies of cytokine characteristics in patients infected with SARS-CoV-2 also showed an increase in Th2 cytokine secretion, which could contribute to lung histopathology [106]. Therefore, the control of the T-cell response should be considered in the development of SARS-CoV-2 vaccines. Proper vitamin D supplementation can mitigate the inflammatory effects of the COVID-19 vaccine.

The vaccine-induced humoral immune response may reflect effective protection against SARS-CoV-2 infection. However, the reaction to aberrant antibodies could have adverse effects in some patients [6]. In SARS-CoV-infected animal models, vaccine-induced S-specific IgG can cause severe acute pulmonary injury since these IgG antibodies disrupted the inflammation resolution response with the blocking of Fc gamma receptor (FcγR) on the cell membrane of activated macrophages [107]. During the acute phase, deceased patients usually display higher levels of neutralizing antibodies (NAbs), which decrease more rapidly than in recovered patients. This reflects the potentially systematic breakdown of the immune system, which causes pathological pulmonary effects [107,108]. Consistently, patients with severe SARS-CoV-2 infections frequently experience significant IgG3 reactions, which were linked to the worst clinical condition with the antibody-dependent enhancement (ADE) of COVID-19 [109]. It is currently unclear whether SARS-CoV-2 vaccines will induce an aberrant reaction to antibodies, and further research is needed to explore potential lung damage from SARS-CoV-2 vaccines. We speculate that appropriate vitamin D supplementation can promote immunity through acceleration and cooperation with IFN-I, promoting the production of antibodies from B cells that are dependent on the T cells of the COVID-19 vaccine.

Age is known to affect vaccine immunity. Vaccinated aged animals that were challenging to immunize also displayed eosinophilic infiltration in the lungs. Neutralizing antibody titers were significantly reduced in aged vaccinated groups compared to young groups [110]. In brief, elderly populations with underlying diseases, including diabetes, hypertension, and cardiovascular disease, are at high risk for vitamin D deficiency and COVID-19 [111]. Given the severity of the disease in elderly people, older animal models are essential for the preclinical validation of vaccines. Even patients on maintenance hemodialysis developed a substantial humoral response following the BNT162b2 vaccine, although it was significantly lower than that of controls. Age was an important factor in the humoral response, regardless of chronic medical conditions [112]. Vit-D and the VDR pathway both have an important anti-inflammatory function, and the lack of vit-D in aged subjects likely increases the risk of chronic mild inflammation conditions [113], resulting in poor responses to vaccines.

Two clinical studies have been presented to explain the potential benefits of vitamin D supplementation for vaccine efficacy. ChAdOx1 nCoV-19 (AZD1222) is a candidate SARS-CoV-2 vaccine comprising a replication-deficient simian adenovirus expressing the whole SARS-CoV-2 spike protein. The vaccine was tolerated, and antigen-specific neutralizing antibodies and T lymphocytes were induced against the SARS-CoV-2 spike protein. Eight weeks after a single-dose vaccination, adults demonstrated an induced S-protein-reactive CD4^+^ T with a T helper (THαβ)-type cytokine bias and CD8^+^ T cells with a cytotoxic phenotype. These are important findings, as THαβ-type immunity is believed to facilitate protective antiviral immunity. Robust B-cell activation and proliferation were also observed, and IgG of anti-S proteins (mainly the IgG1 isotype) were detected from day 14 to day 56. In particular, these antibodies demonstrated neutralizing activity against SARS-CoV-2, and their affinity for the S protein increased from day 28 to day 56. A single vaccination also gave rise to IgM and IgA antibodies specific to the S protein [114]. Besides the antibody titer elevation, ChAdOx1 nCoV-19 vaccination also increased IgG antibody avidity significantly to provide seroprotection. Adequate vit-D can aid in THαβ-type immunity and promote the activation of B cells with higher levels of IgG-neutralizing antibodies. Further investigation of a booster dose of ChAdOx1 nCoV-19 found it to be safe and more tolerable than initiation doses. A study shows that a second vaccination improves the titers of anti-S antibodies and the neutralizing activity, which promotes THαβ-type T-cell responses. Moreover, the booster dose further enhances the functional capacity of anti-S antibodies to support antibody-dependent cellular cytotoxicity, complement deposition, and natural killer cell activation. These have been linked with protective immunity in preclinical studies [115]. All these responses could be accentuated in the presence of vit-D adequacy. Importantly, the second dose of the vaccine was shown to be safe and better tolerated than the first dose. Since the majority of COVID-19 candidate vaccines are designed to target the SARS-CoV-2 spike protein, it remains to be determined whether the specific immunity correlates with vaccine-mediated protection. Thus, this two-dose vaccine regimen is more effective in promoting immunity to SARS-CoV-2 and is well tolerated. These data also suggest that the booster dose should remain effective if administered eight to twelve weeks after the initial vaccination [116]. Similar results should also be noted in other vaccines to show the link between vit-D adequacy and seroconversion or sero-protectivity because vit-D can aid the activation of antiviral THαβ-type immunity.

Better vitamin D status was shown to improve seroconversion in response to influenza vaccinations [82]. The control of the current COVID-19 pandemic and mortality is likely to be highly dependent on effective vaccination, but vitamin D deficiency continues to be common across the U.K. and other nations. Better vitamin D status was also associated with reductions in COVID-19 risks in a prospective study in the USA. Vitamin D supplement is related to a reduction in acute viral respiratory infection. In addition, studies suggest that better vitamin D supplementation in order to correct the deficiency with the metabolite calcifediol can reduce COVID-19 severity and mortality. Inadequate vitamin D serum level is related to COVID-19 incidence, severity, and mortality. The low vitamin D level is shown to be an independent risk factor of SARS-CoV-2 infection and COVID-19 hospitalization. Individuals with vitamin D deficiency tend to have more severe symptoms of SARS-CoV-2 infection. As a result of this information, the correction of vitamin D deficiency is included in the clinical management for the treatment of COVID-19 patients. A recent report using U.K. Biobank data found a strong inverse association of serum 25(OH)D values with COVID-19 severity [117,118].

**Figure 4:**
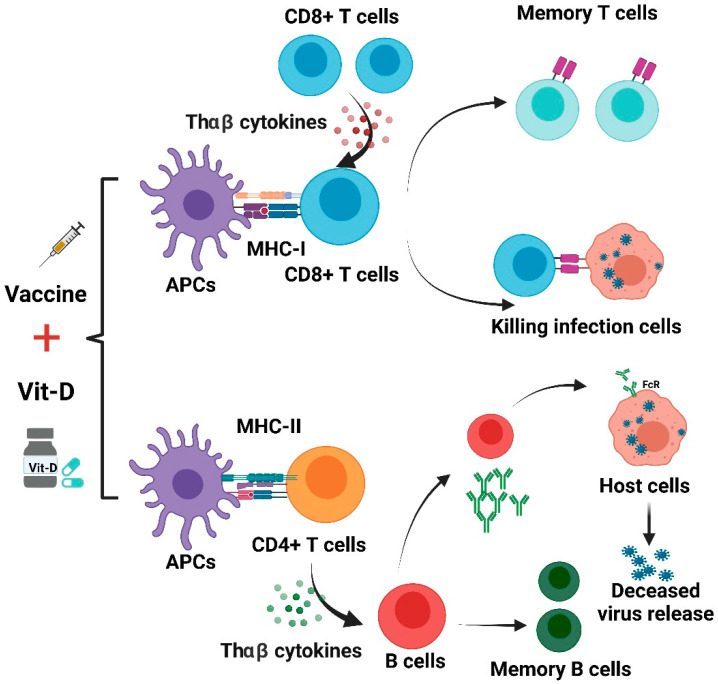
Effects of vitamin D on immune responses induced by COVID-19 vaccines.

We suggest that an intake of vitamin D to reduce the rate of deficiency could provide a simple, safe, and cheap aid in reducing COVID-19 risks. If protection afforded by vaccinations against COVD-19 proved to be increased through the repletion of pre-existing vitamin D deficiencies, these effects would be useful adjunctive measures for reducing COVID-19 risks globally, especially in high-risk groups for COVID-19. A summary of Vit-D effect on COVID-19 vaccines is shown in Figure 4.

The use of vitamin D supplements may improve immune responses from different COVID-19 vaccines. As shown in Figure 4, antigen-presenting cells (APCs) treat the vaccine as an antigen and then present it to CD8+ T and CD4+ T cells. CD8+ T lymphocytes can be activated by THαβ cytokines and acquire the capacity to attack infected cells. This process could be complemented with appropriate vitamin D supplementation. THαβ cytokines can aid in the differentiation of B cells. The activated B cells are able to produce NAbs. Vit-D can also improve antibody generation in a T-cell-dependent B-cell manner. However, unbalanced immune responses can lead to lung immunopathology induced by aberrant ADE, and this may also be mitigated by treatment using vit-D [6,114].

## 9. Conclusions

Vitamin D enhances the innate immunity needed to fight COVID-19 by activating Toll-2 receptors. It also enhances the synthesis of antimicrobial peptides, promotes autophagy through the formation of autophagosomes, and increases the synthesis of lysosomal degradation enzymes in macrophages. In relation to adaptive immunity, vitamin D improves THαβ CD4+ T lymphocytes, suppresses T helper 17 lymphocytes, and promotes the production of interleukin-10 and virus-specific IgG1 antibodies by activating T-dependent B lymphocytes. In addition, vitamin D attenuates the release of proinflammatory cytokines by CD4+ T cells through the signaling of nuclear factor B, thereby inhibiting the development of a cytokine storm. Vitamin D increases the bioavailability and expression of soluble ACE2, which can lead to the entrapment and inactivation of the virus. Vitamin D inhibits renin expression and serves as a negative RAS regulator [47]. Therefore, vitamin D supplements may contribute to the effectiveness of the SARS-CoV-2 vaccine in clinical scenarios. However, this speculation warrants further investigation.

## Data Availability

This is a narrative review article. The primary collection of documents for analysis and review comes from PubMed.
